# Risk factors for pulmonary tuberculosis: a clinic-based case control study in The Gambia

**DOI:** 10.1186/1471-2458-6-156

**Published:** 2006-06-19

**Authors:** Philip C Hill, Dolly Jackson-Sillah, Simon A Donkor, Jacob Otu, Richard A Adegbola, Christian Lienhardt

**Affiliations:** 1 Tuberculosis Division, Medical Research Council Laboratories, Banjul, The Gambia; 2 Programme Tuberculose -UMR 145, Institut de Recherche pour le Développement, Montpellier, France

## Abstract

**Background:**

The tuberculosis (TB) epidemic in Africa is on the rise, even in low-HIV prevalence settings. Few studies have attempted to identify possible reasons for this. We aimed to identify risk factors for pulmonary tuberculosis in those attending a general outpatients clinic in The Gambia, a sub-Saharan African country with relatively low HIV prevalence in the community and in TB patients.

**Methods:**

We conducted a case control study at the Medical Research Council Outpatients' clinic in The Gambia. Pulmonary TB cases were at least 15 years old, controls were age and sex matched clinic attendees. Participants were interviewed using a structured questionnaire.

**Results:**

100 sputum smear positive TB cases and 200 clinic controls were recruited. HIV prevalence was 6.1% in cases and 3.3% in controls. Multivariable assessment of host factors showed that risk of TB was increased among the Jola ethnic group and smokers, and decreased in those in a professional occupation. Assessment of environmental factors showed an increased risk with household crowding, history of household exposure to a known TB case, and absence of a ceiling in the house. In a combined multivariable host-environment model, the risk of TB increased with crowding, exposure to a known TB case, as well as amongst the Jola ethnic group.

**Conclusion:**

In The Gambia, household crowding and past household exposure to a known TB case are the standout risk factors for TB disease. Further research is needed to identify why risk of TB seems to differ according to ethnicity.

## Background

Tuberculosis (TB) causes approximately 2 million deaths per year globally [1]; 98% occur in low-income countries [2]. In Africa, in contrast to many other parts of the world, the incidence rate is rising by approximately 6% per year [3] High rates of HIV infection have been a key driving factor in this [4]. However, even in African countries with relatively low HIV prevalence, the TB case notification has been rising: in The Gambia (where community HIV seroprevalence is approximately 2%[5]) TB case notification rose from 82/100,000 in 1994 to 140/100,000 in 2004 [6]. New ways to tackle the epidemic are urgently needed.

Studies investigating the risk factors for TB have been conducted in a variety of settings, but very few in Africa [7]. Recently, we published a community-based case control study of host and environmental risk factors for TB from 3 West African countries [8] In a combined multivariable model, male gender, HIV infection, smoking, history of asthma, being widowed or divorced, family history of TB, increasing household size and not owning a house were all identified as determinant risk factors for TB.

A particular aspect of conducting case control studies in settings, such as many African countries, is the lack of street address, post-code, telephone or recent reliable census information, that has led investigators to consider various ways to select controls. One approach, as in our previous study, is to select neighbourhood controls using a standard method[9]. However, as we identified, this may lead to overmatching for environmental factors. An alternative approach is to select clinic controls. We searched for host and environmental risk factors for pulmonary TB using a clinic based case control study in The Gambia, where HIV prevalence is low in both the community and in TB patients.

## Methods

The present case control study was conducted at the MRC Laboratories general outpatients' department (OPD), was approved by the joint Gambia government/MRC ethics committee and all study participants provided written informed consent. The MRC Laboratories are located on the edge of a large urban area in the Greater Banjul region in The Gambia. The population of the area is approximately 500,000 people. Patients seeking healthcare at the OPD have a wide variety of conditions, although patients with symptoms strongly suggestive of a surgical or obstetric problem are not seen. The MRC OPD sees approximately 250 patients per weekday and patients can be admitted directly to an onsite ward for hospital care.

Consecutive newly diagnosed sputum smear positive tuberculosis patients older than 15 years were recruited from the OPD. Pulmonary TB was confirmed by two consecutive sputum smears positive for acid-fast bacilli and a positive culture; the smear and culture procedures were conducted as previously described [10].

For each case, two controls were selected. Controls were age (within 10 year age bands) and sex-matched with a respective case. The first 'first-time' OPD attendee fitting the age and sex criteria was approached on a clinic day. If he/she refused to be involved in the study, the next eligible clinic attendee was approached. The control was seen by the study doctor to address their medical complaint and to have a chest x-ray to exclude pulmonary TB. All cases and those controls with a clinical indication, had a blood test, and were counselled to have an HIV test. Those found to be HIV positive were referred for post-test counselling and appropriate care, according to national guidelines.

Study participants answered a structured questionnaire administered in their own language by a trained health worker. Information was collected on a wide range of potential host- and environment- related risk factors for TB, focusing on those that might be amenable to an intervention. Host information collected included basic demographic data (age, gender, ethnicity), past medical history of asthma and diabetes, history of smoking and alcohol consumption, schooling and category of occupation [11] The presence of a BCG scar on the left or right deltoid was checked. Environmental factors considered included the presence of a functional electric or gas cooker, building structure and materials, occupation of the head of the household and whether there had been another member of the household with TB disease. We created a crowding index comprising 3 categories: (1) a household of less than 4 persons and less than 2 people sleeping per room on average; (2) either a household of less than 4 persons and 2 or more persons per room, or a household of at least 4 persons but less than 2 persons per room; (3) a household of at least 4 persons and at least 2 persons per room.

Data were double entered into an ACCESS database and checked for errors. Analysis was conducted using index case and control triplets. Odds ratios (OR) and their 95% confidence intervals (CI), were estimated using conditional logistic regression, with TB as an outcome. The likelihood ratio test used to assess the association between the explanatory variables and the risk of TB, and to test for interaction and trend. Univariable analyses were performed to examine the effect of each variable on the risk of TB. Multivariable models were then constructed, including variables that showed an effect in the prediction of TB in the univariable analyses at the p = 0.05 level of significance. Finally a combined host and environment multivariable model was constructed. The analyses were performed using STATA (version 7, Stata corporation, College Station, TX).

## Results

Over a 2-year period from June 2002 to June 2004, 100 sputum smear and culture positive TB cases plus 200 age and sex matched controls were recruited. Three eligible cases and approximately 20% of eligible controls refused participation. The median age of both cases and controls was 30 years (Table 1). Six (6.1%) of 98 cases tested were HIV positive, compared to 2 (3.3%) of 60 controls tested (p = 0.45). The age, sex and HIV status of the cases recruited from this clinic were similar to those we have previous reported from The Gambia [8]. The most common diagnoses among the controls were gastrointestinal problems (51; 25.5%) and skin complaints (36; 18%). Other diagnoses in at least 5% of controls were: musculoskeletal disorders (16; 8%), cardiovascular disorders (12; 6%), upper respiratory tract infections (13; 6.5%), malaria (13; 6.5%), urinary tract infections (11; 5.5%) and viral illness (10; 5%).

Table 2 shows the assessment of host-related risk factors for TB disease. In the univariable analysis, members of the Jola ethnic group, those widowed or divorced and smokers were significantly more likely to have TB, while trained professional workers (eg. school teacher, nurse, doctor, pharmacist) had significantly lower risk. In the multivariable analysis, Jola (p = 0.028) and smoking (p = 0.032) remained significant risk factors, while professional workers also remained at significantly reduced risk (p = 0.039).

Table 3 shows the assessment of environmental risk factors for TB disease. In the univariable analysis, the absence of a ceiling, walls made of mud, the highest category of household crowding, and a history of TB in another member of the household were all found to be associated with TB disease. In a multivariable model, the absence of a ceiling (p = 0.032), household crowding (likelihood ratio test for linear trend: p = 0.0013) and a history of TB in another household member (p < 0.0001) remained significant.

Table 4 shows the results of a multivariable model assessing both host and environmental risk factors that were significant in their respective individual analyses. Being in the Jola ethnic group (p = 0.012), the highest crowding category (p = 0.003) and a history of TB in another household member (p < 0.0001) remained significant risk factors. Increasing crowding across the three categories was significantly associated with TB by the likelihood ratio test for linear trend (p = 0.0038). The absence of a ceiling remained significant only at the p = 0.1 level of significance, whereas smoking and occupation lost significance in this combined model. No significant interactions between variables were found.

## Discussion

In this clinic-based case-control study we have identified key risk factors for tuberculosis in The Gambia. Overcrowding and a history of household exposure to a known TB case are the standout risk factors in this setting, while we also found that being in the Jola ethnic group was a risk factor.

The finding that household exposure to a known TB case is by far the most important risk factor for TB in this TB-endemic setting is consistent with our previous study, where 24% of West African TB cases had a family history of TB compared to 10% of controls (OR 3.24; 95% CI 2.3–4.6; p < 0.001)[8]. Here, 45% of cases reported household exposure to a known TB case, compared to 11% of controls. This finding is of substantial public health importance. Increased household size was found to be important in our previous study, and overcrowding has been documented as a risk factor for TB from several other studies in a variety of settings [12,13]. It is of note that three quarters of cases and 60% of controls in this study were from households that were in the highest crowding category-reflecting the extent of this issue in urban Gambia.

Differences in TB rates between racial groups have been previously described. It is thought that a large proportion of the differences seen can ultimately be explained by environmental and behavioural factors [14,15]. Our finding in Jola remained significant despite adjustment for a number of host and environmental factors. However, this finding could be due to other factors that have not been considered, such as a difference in geographical residence of the various ethnic groups covered by our clinic, leading to a differential distribution of the time to attend the clinic, and thus a lower chance for Jola persons to be selected as controls.

In the combined multivariate analysis smoking was not an independent risk factor for TB. However, in previous case-control studies, we and others found that smoking is a risk factor and that there is strong dose-response relationship between smoking and TB [8,16,17]. In this study we sought to identify those who were smokers only during the previous 6 months. By doing so, we may have underestimated the effect of smoking. Furthermore, while selection of clinic controls is reasonable when the source population is difficult to define, bias can be introduced if any controls are admitted because of an illness that is related to a risk factor under study. Smoking related diseases are obvious candidates for this bias, leading to an underestimate of the effect of smoking with respect to the disease under study. While the absence of a ceiling was only significant at the p = 0.1 level of significance in the combined model, it does stand in contrast to our previous finding in the community based study that showed, in the univariable analysis, a trend in the opposite direction of borderline significance (OR 0.79; 0.63–1.00; p = 0.05) [8]. There is no obvious explanation for this difference.

A potential weakness in our study is that less than a third of controls had an HIV test. The study was considered to not have the power to assess HIV infection as a risk factor for disease, noting our previously reported prevalence in sputum smear positive cases of 8% [18], and in the community of approximately 2%[5]. To address this issue we have re-run our analyses excluding those that are known to be HIV positive and our findings were not significantly altered. A further issue is a potential bias in this community associated with questions about asthma and diabetes-certainly mild forms of these diseases may well have not been diagnosed. Further, because of the unreliability of verbal history, we did not ask about a history of worms or worm treatment. It is also worth noting that, because of matching, we could not assess the importance of increased age and male sex, both factors having been shown previously to be associated with TB [7,19,20].

## Conclusion

With respect to opportunities for intervention, this study has identified several possibilities. First of all, since a history of a known TB contact inside the household is extremely common in The Gambia for those who develop disease themselves, active tracing of TB case contacts should be considered to identify co-prevalent cases and to encourage early attendance at TB clinics for those who have symptoms in the long term. Second, ways to avoid overcrowding in urban settings should be sought. There is no easy solution to this problem, as it is inextricably linked to indices of wealth at a population level. Third, further studies are required to ascertain whether the Jola ethnic group have a behavioural cause for their increased rate of TB, which may be amenable to intervention. Finally, while smoking was not an independent risk factor in a final combined model, there is ample evidence from other studies to confirm it as a risk factor for TB disease and it is a known killer of its own right.

## Competing interests

The author(s) declare that they have no competing interests.

## Authors' contributions

PH was involved in the design of the study, supervised the field work and drafted the manuscript. DJS was involved in the design of the study, conducted the field work and assisted in the data analysis and write-up. SD managed the data entry and verification and quality and contributed to the write-up. RA supervised the microbiological aspects of the study with JO, and contributed to the write-up. CL was involved in the design of the study, analysis and write-up. All authors read and approved the final manuscript.

## Pre-publication history

The pre-publication history for this paper can be accessed here:



## Figures and Tables

**Table 1:** Characteristics of TB cases and clinic controls

	**Cases (n = 100)**	**Controls (n = 200)**
Age, years		
Mean (std dev)	33.6 (14.4)	33.5 (13.2)
Median (range)	30 (15–75)	30 (15–75)
Sex, n (%)		
Male	65 (65)	130 (65)
Female	35 (35)	70 (35)
HIV positive, n(%)	6^a ^(6.1)	2^b ^(3.3)

^a ^n = 98, ^b ^n = 60

**Table 2:** Host-related factors for tuberculosis: comparison of TB cases and clinic controls.

**Variable**	**Controls**	**Cases**	**Total No. triplets**	**OR (95% CI)**	**p-value**	**Adj^a^OR (95% CI)**	**Adj^a ^p value**
Ethnic group, n(%)			100				
Mandinka	105 (52.5)	41 (41)		1.0		1.0	
Wolof	25 (12.5)	8 (8)		1.25 (0.48–3.25)	0.65	1.45 (0.54–3.89)	0.42
Fula	28 (14)	11 (11)		0.94 (0.42–2.10)	0.87	1.06 (0.46–2.44)	0.89
Jola	21 (10.5)	22 (22)		2.70 (1.33–5.48)	0.006	2.41 (1.10–5.32)	0.028
Other	31 (15.5)	18 (18)		1.53 (0.75–3.10)	0.24	1.79 (0.84–3.84)	0.133
Marital status, n(%)			100				
Married	118 (59)	48 (48)		1.0		1.0	
Single	74 (37)	43 (43)		1.58 (0.86–2.90)	0.14	1.46 (0.68–3.12)	0.33
Widowed/divorced	8 (4)	9 (9)		3.19 (1.02–9.93)	0.046	3.04 (0.86–10.75	0.085
Smoker in past 6 months			100				
No	167 (83.5)	72 (72)		1.0		1.0	
Yes	33 (16.5)	28 (28)		2.37 (1.22–4.63)	0.011	2.22 (1.07–4.59)	0.032
Alcohol							
Never	189 (94.5)	90 (90)		1.0			
Current/past	11 (5.5)	10 (10)		2.05 (0.79–5.32)	0.14		
BCG scar			100				
No/unsure	143 (71.5)	73 (73)		1.0			
Yes	57 (28,5)	27 (27)		0.93 (0.54–1.60)	0.78		
History of asthma			100				
No	198 (98)	99 (99)		1.0			
yes	2 (1)	1 (1)		1.0 (0.09–11.03)	1.0		
History of diabetes			100				
No	200 (100)	97 (97)		-	-		
Yes	0 (0)	3 (3)					
Years of schooling			100				
None	42 (21)	25 (25)		1.0			
1–4	24 (12)	10 (10)		0.65 (0.26–1.62)	0.36		
5–8	40 (20)	27 (27)		1.07 (0.52–2.22)	0.85		
>8	94 (47)	38 (38)		0.60 (0.30–1.22)	0.16		
Occupation			100				
Unemployed	16 (8)	10 (10)		0.89 (0.35–2.28)	0.81	1.01 (0.37–2.75)	0.98
Unskilled worker	29 (14.5)	22 (22)		1.0			
Skilled worker	96 (48)	41 (41)		0.51 (0.25–1.02)	0.055	0.62 (0.29–1.33)	0.22
Professional	22 (11)	3 (3)		0.15 (0.04–0.60)	0.007	0.24 (0.06–0.93)	0.039
Other	27 (18.5)	24 (24)		0.94 (0.41–2.14	0.89	1.48 (0.57–3.87)	0.42

^a^Adjusted through multivariable analysis

**Table 3:** Environmental factors for tuberculosis: comparison of TB cases and clinic controls.

**Variable**	**Controls n (%)**	**Cases n (%)**	**Total No. triplets**	**OR (95% CI)**	**p-value**	**Adj^a ^OR (95% CI)**	**Adj^a ^p value**
Electric or gas cooker			100				
Absent	159 (79.5)	86 (86%)		1.0			
Present	41 (20.5)	14 (14%)		0.60 (0.29–1.21)	0.15		
Ceiling			100				
Yes	155 (77.5)	63 (63)		1.0			
No	45 (22.5)	37 (37)		2.08 (1.21–3.57)	0.008	2.27 (1.07–4.83)	0.032
Walls			100				
Cement	136 (68)	56 (56)		1.0			
Mud	4 (2)	11 (11)		5.92 (1.86–18.81)	0.003	4.52 (0.93–21.98)	0.062
Mudbrick/other	60 (30)	33 (33)		1.30 (0.77–2.19)	0.33	0.83 (0.39–1.74)	0.62
Floor			100				
Earth	27 (13.5)	18 (18)		1.0			
Concrete/tiles	170 (85)	71 (71)		0.72 (0.39–1.37)	0.32		
Number of windows			99				
0–1	60 (30.3)	29 (29.3)		1.0			
2–4	116 (58.5)	51 (51.5)		0.89 (0.51–1.54)	0.67		
>4	24 (12.1)	18 (18.2)		1.49 (0.69–3.23)	0.32		
Occupation head of house			74				
Unemployed	18 (10.2)	10 (12.5)		0.55 (0.19–1.55)	0.26		
Unskilled worker	37 (21.0	10 (12.5)		1.0			
Skilled worker	18 (10.2)	3 (3.8)		0.96 (0.37–2.48)	0.94		
Professional	86 (48.8)	40 (50)		0.33 (0.07–1.46)	0.14		
Other	17 (9.7)	17 (21.3)		1.95 (0.63–5.96)	0.24		
Crowding index			91				
1	44 (23.2)	10 (11.8)		1.0			
2	31 (16.4)	11 (12.9)		1.49 (0.54–4.09)	0.44	2.39(0.71–8.02)	0.16
3	189 (60.3)	64 (75.3)		2.31 (1.05–5.12)	0.038	5.12 (1.82–14.38)	0.002
Household member had TB			100				
No/unsure	178 (89)	55 (55)					
yes	22 (11)	45 (45)		6.02 (3.23–11.25)	<0.0001	7.55 (3.43–16.6)	<0.0001

^a^Adjusted through multivariable analysis

**Table 4:** Combined multivariable assessment of host and Environmental factors for tuberculosis: comparison of TB cases and clinic controls (n = 274).

**Variable**	**Adj^a ^OR (95% CI)**	**Adj^a ^p value**
Ethnic group, n(%)		
Mandinka	1.0	
Wolof	2.76 (0.65–2.01)	0.17
Fula	0.78 (023–2.64)	0.70
Jola	3.83 (1.35–10.86)	0.012
Other	0.94 (0.32–2.74)	0.91
Smoker in past 6 months		
No	1.0	
Yes	1.88 (0.83–4.26)	0.13
Occupation		
Unemployed	2.70(0.56–12.94)	0.22
Unskilled worker	1.0	
Skilled worker	0.65 (0.24–1.71)	0.38
Professional	0.22 (0.03–1.72)	0.15
Other	1.17 (0.38–3.61)	0.79
Ceiling		
Yes	1.0	
No	2.12 (0.91–4.93)	0.082
Crowding index		
1	1.0	
2	2.58 (0.69–9.68)	0.16
3	5.05 (1.75–14.62)	0.003
Household member had TB		
No/unsure		
Yes	10.17 (4.08–25.63)	<0.0001

^a^Adjusted
